# Distinct Ecological Habits and Habitat Responses to Future Climate Change in Two Subspecies of *Magnolia sieboldii* K. Koch, a Tree Endemic to East Asia

**DOI:** 10.3390/plants13213097

**Published:** 2024-11-03

**Authors:** Meng Li, Chang-Fei Zheng, Xiang-Qian Gao, Chang-Hong Li, Yong-Xiang Li, Xin-He Xia, Jun Yang, Yong-Qi Zheng, Ping Huang

**Affiliations:** 1 State Key Laboratory of Tree Genetics and Breeding, Chinese Academy of Forestry, Beijing 100091, China; limengde11@163.com (M.L.); changfeizheng@163.com (C.-F.Z.); gaoxq@caf.ac.cn (X.-Q.G.); lichanghong@caf.ac.cn (C.-H.L.); lyxiang97@163.com (Y.-X.L.); xinhex355@163.com (X.-H.X.); 2 Laboratory of Forest Silviculture and Tree Cultivation, National Forestry and Grassland Administration, Research Institute of Forestry, Chinese Academy of Forestry, Beijing 100091, China; 3 Jiangxi Wuyuan National Nature Reserve of Forest Birds, Shangrao 333200, China; lyckyj@163.com

**Keywords:** *Magnolia sieboldii*, subspecies, climate change, MaxEnt model, potential habitat distribution, conservation

## Abstract

*Magnolia sieboldii*, an important ornamental tree native to East Asia, comprises two subspecies in distinct regions, with wild populations facing suboptimal survival. This study aimed to understand the potential habitat distribution of these subspecies under future climate-change conditions to support climate-adaptive conservation. The maximum entropy (MaxEnt) model was used with occurrence and environmental data to simulate the current and future suitable habitats under various climate scenarios. Precipitation in the warmest quarter played a crucial role in shaping the potential habitats of both subspecies; however, they exhibited different sensitivities to temperature-related variables and altitude. *Magnolia sieboldii* subsp. *sieboldii* is more sensitive to temperature seasonality and annual mean temperature, whereas *Magnolia sieboldii* subsp. *japonica* is more affected by altitude, mean temperature in the driest quarter, and isothermality. Currently, the subsp. *sieboldii* is predicted to have larger, more contiguous suitable habitats across northeastern China, the Korean Peninsula, and Japan, whereas the subsp. *japonica* occupies smaller, more disjunct habitats scattered in central and western Japan and the southern Chinese mountains. These two subspecies will respond differently to future climate change. Potentially suitable habitats for subsp. *sieboldii* are expected to expand significantly northward over time, especially under the SSP585 scenario compared with the SSP126 scenario. In contrast, moderately and highly suitable habitats for the subsp. *japonica* are projected to contract southward significantly. Therefore, we recommend prioritizing the conservation of the subsp. *japonica* over that of the subsp. *sieboldii*. Strategies include in situ and ex situ protection, introduction and cultivation, regional hybridization, and international cooperation. Our study offers valuable insights for the development of targeted conservation strategies for both subspecies of *M. sieboldii* to counteract the effects of climate change.

## 1. Introduction

Climatic factors are the primary environmental determinants that influence the regional-scale geographic distribution of plants [1]. According to the latest assessment report (AR6) of the Intergovernmental Panel on Climate Change (IPCC), global surface temperatures are projected to continue rising until at least the mid-century. Global warming affects plant growth and development across the entire plant life cycle, decreases the fitness of many wild plant species in their natural habitats [2], and triggers changes in species distribution worldwide [3]. Consequently, a significant portion of the flora worldwide is anticipated to become extinct within this century [3,4]. Climate change is a primary threat to global biodiversity. Therefore, predicting the potential distribution of suitable habitats for species and their future migration trends in response to climate change can offer valuable scientific insights into habitat management and plant conservation.

*Magnolia sieboldii* K. Koch is a deciduous shrub to small tree that is endemic to East Asia belonging to the genus *Magnolia* of the family Magnoliaceae. This rare ornamental plant is prized for its large, fragrant, and pure white flowers, described as “probably the purest white of all the magnolia flowers” [5], and its ripe fruits, which are purplish-red. Moreover, its flowers, leaves, and seeds contain an aromatic oil with excellent permeability and long-lasting fragrance, making it a suitable high-quality spice [6], with a seed oil content of up to 39.03% [7]. Furthermore, *M. sieboldii* has a natural distribution extending up to 41° N, and exhibits robust cold resistance. This makes it a valuable hardy germplasm resource, given that most species within the Magnoliaceae family are distributed in subtropical and tropical regions, making them susceptible to cold temperatures. Additionally, many of these species can undergo interspecific hybridization with *M. sieboldii*, including *M. virginiana*, *M. grandiflora, M. insignis, M. tripetala, M. yuyuanensis*, and *M. hypoleuca* [8,9,10]. However, the survival status of wild *M. sieboldii* populations is not optimistic, and it has been listed as a vulnerable species in the “Red List of Chinese Species” [11] because of the clear trend of population decline [12,13]. According to our field survey, this species is mainly distributed in mountainous regions, with populations at higher altitudes in lower latitudes. Mountainous plants are more sensitive to climate change because of their narrow habitat tolerance, and they have a higher extinction risk [3]. Therefore, there is an urgent need to predict the current potential habitat distribution and assess the impacts of future climate change on it to protect this valuable germplasm.

The most common approach for predicting the potential geographical distribution of species and how their ranges shift with climate change is to construct correlative species distribution models (SDMs) [14]. Maximum entropy (MaxEnt) modeling has contributed significantly to the prediction of biodiversity loss under future climate scenarios and is considered one of the most reliable SDMs [15]. One core assumption of SDMs is that all populations respond uniformly to the climate [14]. Therefore, when assessing the impact of climate change on species, most models perform simulations at the species level, often neglecting within-species variations and local adaptations [16]. However, species are not uniform entities but are composed of different populations, are sometimes locally adapted, and exhibit varying levels of plasticity [17]. Therefore, this assumption may not apply to all species, particularly those that have undergone subspecies differentiation. Subspecies represent intraspecific subgroups that are influenced by the local environment in which they are distributed, resulting in morphological or physiological differences. Numerous studies have compared the SDMs constructed at the subspecies and species levels and found significant differences in the results. Subspecies models outperform species models in terms of species distribution, accuracy, realism, and quality [14,18,19,20].

*M. sieboldii* was classified into two subspecies by the Japanese scholar Kunihiko Ueda (1980) based on morphological variations and differences in geographical distribution [21]. *Magnolia sieboldii* subsp. *sieboldii* is more frequent and distributed over the Korean Peninsula and Northeast China, whereas *Magnolia sieboldii* subsp. *japonica* is very rare and found in western Japan and southeastern China [22]. These two subspecies differ in their habits, habitats, and morphological features [21] and niche identity tests have confirmed the divergence of their niches [23]. A previous study conducted distribution simulations for both subspecies during past periods (the Last Glacial Maximum and the Last Interglacial) as well as the present, revealing that their potential distribution areas are relatively independent in the current period [23]. Both subspecies maintained a constant latitudinal distribution throughout the last glacial cycle via migration to lower altitudes, and the mean altitude of suitable habitats (grid cells) for both subspecies shifted upward from the Last Glacial Maximum to the present [23]. In studies concerning the response of species distribution to future climate change, some have found that different subspecies or populations in different distribution areas of a species may exhibit different trends of change, mostly in the horizontal direction [16,24]. In the context of an overall gradual warming climate in the future, it will be of great interest to explore how the range and area of the potential distribution zones of the two subspecies of *M. sieboldii* will respond.

Based on this understanding, we independently constructed MaxEnt models for each subspecies to obtain more accurate and reliable prediction results, improve our understanding of their habitat preferences and responses to climate change, and provide appropriate and targeted conservation recommendations. Based on the occurrence and environmental data of the two subspecies, ArcGIS v.10.4.1 software and the MaxEnt model were used to simulate their current (1970–2000) and future (2041–2060, 2081–2100) suitable habitats under different climate scenarios (SSP126 and SSP585) to (1) predict the distribution pattern of the potentially suitable habitats of the two subspecies under current climatic conditions; (2) identify the key environmental factors affecting the distribution of the two subspecies and quantitatively describe the environmental conditions suitable for their survival; and (3) explore whether the responses of the two subspecies to climate change are consistent, reveal the habitat redistribution pattern of the two subspecies under future climate change, and identify the hotspots of habitat degradation and expansion in order to provide appropriate and targeted theoretical support for the conservation and management of the two subspecies of *M. sieboldii*.

## 2. Results

### 2.1. Optimal Parameters and Accuracy Evaluation of Model

For the subsp. *japonica*, the optimal model parameters included a regularization multiplier (RM) of 1.3 and a feature combination (FC) of threshold features (Ts). For the subsp. *sieboldii*, the optimal model parameters were a RM of 0.1 and a FC consisting of quadratic features (Qs) and product features (Ps).

**Figure 1:**
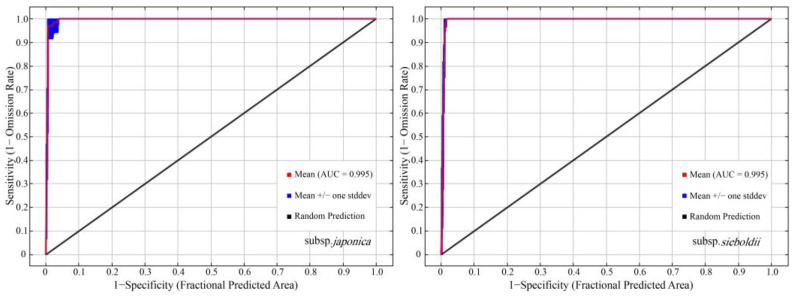
Receiver operating characteristic (ROC) curve tests for the accuracy of model prediction.

We used the optimized parameters to construct MaxEnt models for the two subspecies and obtained the receiver operating characteristic (ROC) curves. The area under the curve (AUC) values were 0.995 for the subsp. *japonica* and 0.994 for the subsp. *sieboldii* (Figure 1), both exceeding 0.9, indicating high predictive accuracy [25].

### 2.2. Dominant Environmental Variables Influencing the Potential Geographical Distribution of Two Subspecies

**Figure 2:**
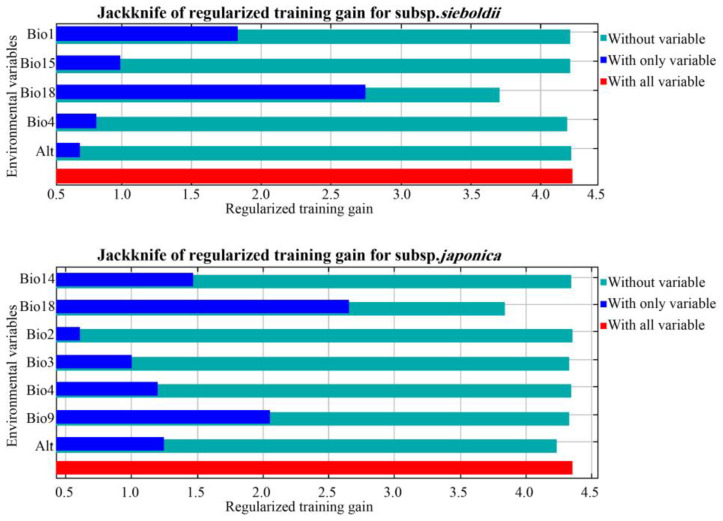
Results of the variable-importance jackknife test.

Based on the percentage contributions and permutation importance of the environmental variables derived from the model (Table 1), for the subsp. *sieboldii*, the top three environmental variables contributing to the model were Bio4 (temperature seasonality), Bio18 (precipitation of the warmest quarter), and Bio1 (annual mean temperature), accounting for 98.7% of the model’s contribution. Similarly, these variables exhibited the highest permutation importance (97.4%). The jackknife test indicated that Bio18 and Bio1 had the greatest individual gains, and omitting Bio18 significantly reduced model performance (Figure 2). Overall, Bio18, Bio4, and Bio1 are the dominant factors influencing habitat distribution of the subsp. *sieboldii*. For the subsp. *japonica*, Bio18 was the most influential, contributing 61.8% to the model (Table 1), followed by Alt (altitude), Bio3 (isothermality), and Bio9 (mean temperature of the driest quarter). Together, these four variables accounted for 91.7% of the contribution. Bio18 also had the highest permutation importance (90.4%) and the jackknife test confirmed its critical role (Figure 2). Collectively, Bio18, Alt, Bio9, and Bio3 were the key drivers of habitat suitability for the subsp. *japonica*.

The response curve of the environmental variables reflects the relationship between environmental variables and habitat suitability [26]. This study used an occurrence probability threshold of *p* ≥ 0.3 to determine the dominant environmental variables’ suitability. For the subsp. *sieboldii*, optimal conditions included precipitation during the warmest quarter ranging from 473.2 to 855.5 mm (peak at 748.2 mm) (Supplementary Figure S1; Table 2), mean annual temperature between 4.4 and 18.2 °C (peak at 11.2 °C), and temperature seasonality ranging from 850.6 to 1351.8 (peak at 1186.6). For the subsp. *japonica*, favorable conditions encompassed precipitation in the warmest quarter ≥ 529.5 mm (optimum 836.8 mm) (Supplementary Figure S2; Table 2), altitude from 709 to 1776.3 m (optimum 1310.7 m), mean temperature of the driest quarter between −5.3 and 6.0 °C (optimum 4.7 °C), and isothermality between 23.2 and 29.7 (optimum 27.3).

### 2.3. Current Distribution of Potentially Suitable Habitats for the Two Subspecies

**Figure 3:**
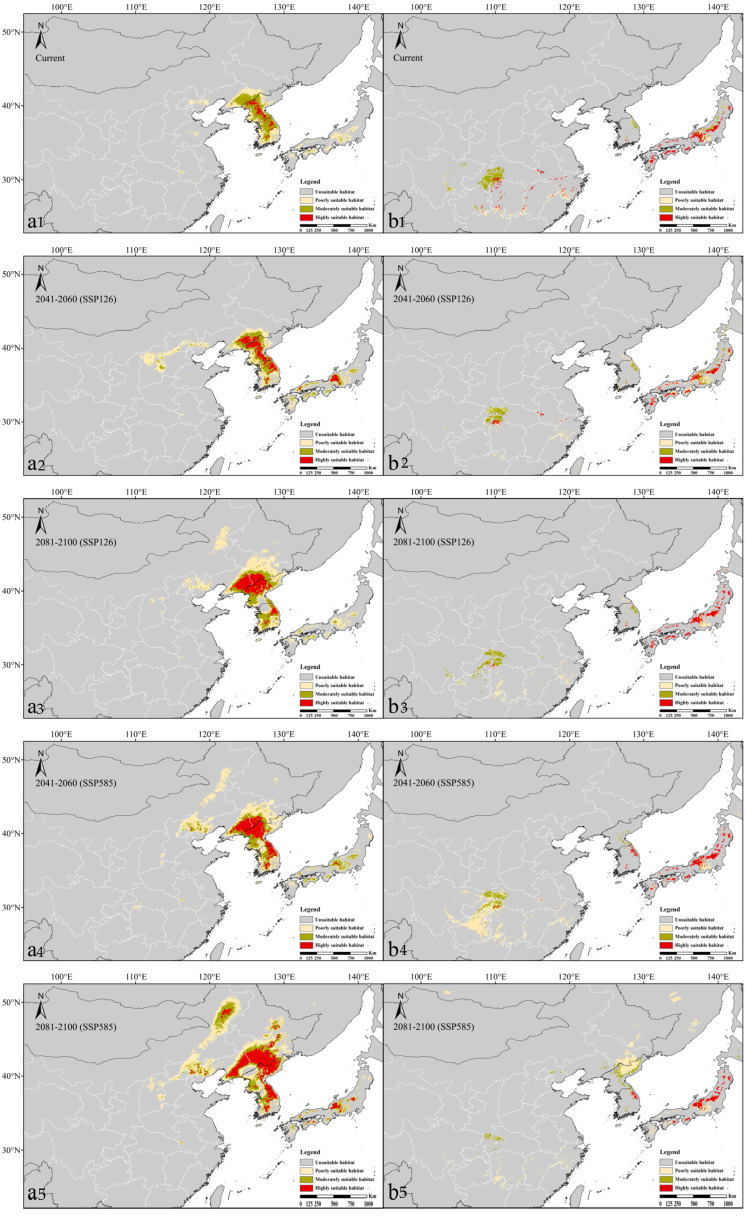
Distribution of the potentially suitable habitats of the two subspecies under different climate scenarios in the present and future periods ((**a1–a5**) subsp. *sieboldii* and (**b1–b5**) subsp. *japonica*).

Under the current climatic conditions, the potentially suitable habitat for the subsp. *sieboldii* was mainly concentrated in East Asia (Figure 3), including China, the Korean Peninsula, and Japan, with a total area of 20.42 × 10^4^ km^2^ (Supplementary Table S1). The highly suitable habitat covered only 1.52 × 10^4^ km^2^ and was concentrated in the central part of the Korean Peninsula, with sporadic distribution in the eastern part of Liaoning, China, which was consistent with the actual occurrence records. The moderately suitable habitat (7.51 × 10^4^ km^2^) and poorly suitable habitat (11.40 × 10^4^ km^2^) were mainly distributed around the highly suitable habitat, as well as Kyushu Island of Japan and the Hebei, Shandong, Anhui, and Zhejiang Provinces of China.

For the subsp. *japonica*, the total potentially suitable habitat was smaller (11.20 × 10^4^ km^2^) but similarly concentrated in China, Korea, and Japan. The highly suitable areas (3.40 × 10^4^ km^2^) were scattered across Japan and Southern China, while the moderately suitable habitat (4.67 × 10^4^ km^2^) surrounded these areas, notably forming a large continuous region along the border of the Chongqing and Hubei Provinces in China, where no occurrence records exist. Poorly suitable habitat (3.13 × 10^4^ km^2^) was found around moderately and highly suitable zones. Compared with the subsp. *sieboldii,* the suitable habitats of the subsp. *japonica* were smaller and more fragmented.

### 2.4. Changes in the Potentially Suitable Habitats of Two Subspecies Under Future Climate Scenarios

**Figure 4:**
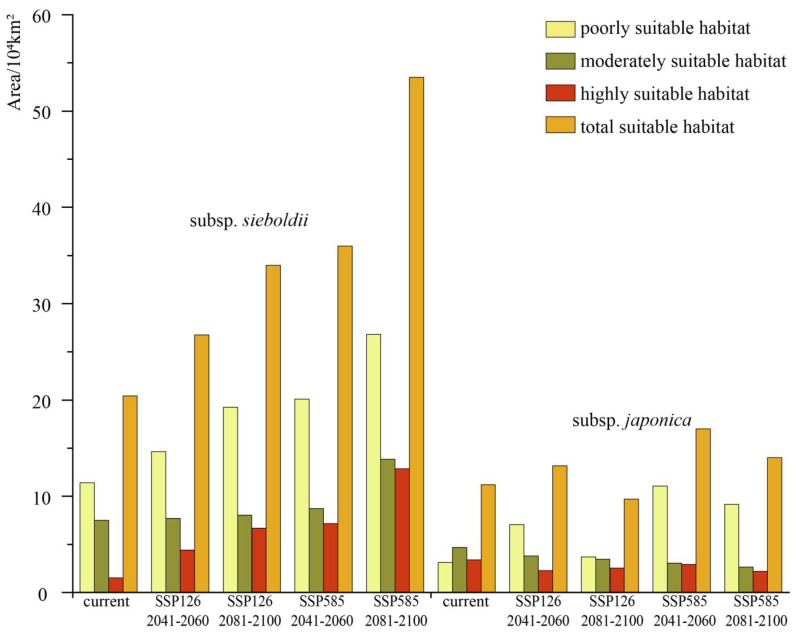
Changes in the area of the potentially suitable habitats of the two subspecies in different periods under two climate scenarios.

For the subsp. *sieboldii*, under scenario SSP126, the total areas of potentially suitable habitat in the two future periods (2041–2060 and 2081–2100) were 26.75 × 10^4^ km^2^ and 33.99 × 10^4^ km^2^, respectively, representing increases of 30.99% and 66.44% compared with the current period (Supplementary Table S1). The trends of highly, moderately, and poorly suitable habitats were consistent with those of total suitable habitats, indicating a continuous increase in the future (Figure 4). Among these, highly suitable habitats exhibited the most significant increases, reaching 191.14% and 341.55% in the two future periods, respectively. They gradually expanded northward from the Korean Peninsula to the border between China and North Korea by 2081–2100 (Figure 3).

Under scenario SSP585, highly, moderately, and poorly suitable habitats, as well as the total suitable habitat of the subsp. *sieboldii*, exhibited a consistent trend of increasing area and gradually expanding northward in the two future periods (Figure 4), similar to that observed under scenario SSP126. Notably, in both periods, the areas of each suitable habitat under SSP585 were larger than their corresponding values under SSP126; in fact, they were approximately twice as large (except for the moderately suitable habitat).

For the subsp. *japonica*, under scenario SSP126, the total potentially suitable habitat in the two future periods was projected to be 13.15 × 10^4^ km^2^ and 9.72 × 10^4^ km^2^, respectively, representing an increase of 17.42% and decrease of 13.21% compared with the current period (Supplementary Table S1). The highly suitable habitat areas were 2.27 × 10^4^ km^2^ and 2.54 × 10^4^ km^2^, representing decreases of 33.33% and 25.26%, respectively. By 2081–2100, highly suitable habitats in China were projected to disappear, except in the Anhui, Zhejiang, and Hubei Provinces (Figure 3). However, a slight expansion was expected in northeastern South Korea, northeastern Honshu Island, and southwestern Hokkaido, Japan. Moderately suitable habitats were also expected to decrease in the future.

Under scenario SSP585, both highly and moderately suitable habitats showed a consistent decreasing trend during the two future periods (Figure 4). Notably, by 2081–2100, highly suitable habitat will retreat to the northeast of Honshu Island in Japan and Republic of Korea, whereas in China, it will completely disappear (Figure 3). Meanwhile, poorly and moderately suitable habitats expanded extensively in the Jilin and Heilongjiang Provinces of China, North Korea, and Russia’s Far East.

### 2.5. Shift in the Centroids of Highly Suitable Habitats Under Two Future Climate Scenarios

**Figure 5:**
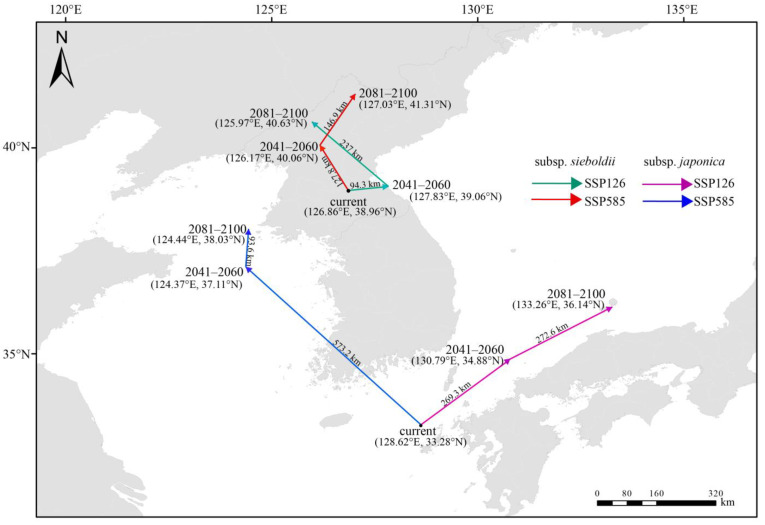
Shifting centroids of highly suitable habitat under two future climate scenarios.

Under current climatic conditions, the centroid of the highly suitable habitat for the subsp. *sieboldii* was located in Southern North Korea (126.86° E, 38.96° N). Under both the SSP126 and SSP585 scenarios, the centroid migrated northward over a greater migration distance under scenario SSP585 (Figure 5). The centroid of the highly suitable habitat for the subsp. *japonica* was located in southwestern Japan (128.62° E, 33.28° N). Under both scenarios, the centroid moved to higher latitudes. However, under scenario SSP126, it shifted northwest, whereas under scenario SSP585, it shifted northeast. This demonstrates the complex impacts of climate change and uncertainty in biological responses.

## 3. Discussion

### 3.1. Dominant Environmental Factors Affecting the Distribution of Two Subspecies

Climate factors constrain the spatial distribution of species at the regional scale, with hydrothermal conditions playing a dominant role [27]. In this study, the habitat distribution of the subsp. *sieboldii* was primarily influenced by precipitation in the warmest quarter (Bio18), temperature seasonality (Bio4), and annual mean temperature (Bio1). For the subsp. *japonica*, habitat distribution was mainly affected by precipitation in the warmest quarter (Bio18), altitude (Alt), average temperature in the driest quarter (Bio9), and isothermality (Bio3). A previous study simulated the currently suitable habitat for *M. sieboldii* in China at the species level and found that the annual temperature range (Bio7) and precipitation in the wettest quarter (Bio16) were the dominant environmental variables [28], a result similar to ours. The seeds of *M. sieboldii* exhibit morpho-physiological dormancy, characterized by an immature embryo that requires a prolonged period of low-temperature stratification to break physiological dormancy and complete morphological development before germination [29,30]; proper temperature and humidity are important in this process. This suggests a physiological limit to the distribution of the two subspecies of *M. sieboldii*.

*M. sieboldii* has an ecological preference for warm and cool conditions, and precipitation during the summer half of the year significantly influences its distribution [31]. Therefore, precipitation in the warmest quarter played a dominant role in the habitat distribution of both subspecies, but the subsp. *sieboldii* showed suitability for survival in the range of 473.2–855.5 mm, with the optimum value at 748.2 mm; and the subsp. *japonica* demonstrated suitability for survival in the range of ≥529.5 mm, with the optimum value at 836.8 mm (Table 2). The latter exhibited a significantly higher demand for precipitation during the warm season. This indicates that the two subspecies within the same species exhibited similar ecological requirements and some degree of niche differentiation, as demonstrated in a previous study [23]. This differentiation is likely driven by long-term environmental adaptations to different climatic regions. The subsp. *sieboldii* is distributed in temperate monsoon climatic regions with four distinct seasons, significant seasonal changes, and large annual temperature ranges. Summers are hot and winters are cold. Thus, it is sensitive to temperature seasonality and annual mean temperatures. The subsp. *japonica* is distributed in subtropical monsoon regions with a small annual temperature range, high temperatures in summer, mild temperatures in winter, and humid conditions throughout the year. Thus, it is sensitive to the altitude, average temperature in the driest quarter, and isothermality.

### 3.2. Potentially Suitable Habitat in Current Climate Condition

A previous study of *M. sieboldii* at the species level revealed that highly suitable habitats (*p* > 0.48) were mainly concentrated in two regions [28]. One region was located in the southern part of Jilin Province and the eastern part of Liaoning Province, which is generally consistent with the positions of moderately and highly suitable habitats (*p* > 0.4) in China for the subsp. *sieboldii* in this study. The other region was located at the junction of the Anhui, Zhejiang, Jiangxi, and Fujian Provinces, diverging from the simulation results for the subsp. *japonica* presented in this study. Our study indicated that only moderately and highly suitable small habitats are scattered throughout this area. Notably, the simulation results from our study closely correspond to the actual distribution of the species. There were substantial differences between the simulation results of subspecies and species models, with both realism and model quality being superior in the former [14,18,19,20]. The authenticity of the species occurrence data, their coverage of the species range, and combination of different environmental variables all influence simulation results [32,33,34]. In contrast to the aforementioned study on *M. sieboldii* [28], the occurrence records used in this study were largely obtained from field surveys, covering the distribution ranges of both subspecies and excluding unreliable records in regions such as Henan and Sichuan Provinces. However, the environmental variables used to construct the MaxEnt model only included climatic variables and altitude, neglecting other environmental factors, such as soil. Although climatic factors play a vital role in determining the regional-scale geographical distribution of plants, species distribution is also constrained by various environmental variables such as soil and topography [35]. Thus, the differences in the simulation results may be related to modeling at the subspecies level or may stem from disparities in species occurrence records and the environmental variables employed in MaxEnt.

Under the current climatic conditions, the area of potentially suitable habitats for the subsp. *japonica* is significantly smaller than that for the subsp. *sieboldii* (Figure 4) and exhibits a patchy and fragmented distribution (Figure 3). This finding is supported by Ueda [21] and Murata et al. [22], who noted that the subsp. *sieboldii* is common in North Korea. It grows as a large shrub or small tree, thrives vigorously in hilly to montane regions at elevations of 100–1500 m, and typically dominates various habitats such as stream sides, warm-to-cool temperate deciduous forests, and occasionally dry *Pinus* forests, as well as slopes or ridges. The subsp. *sieboldii* is hardier and easier to cultivate at lower elevations in Japan and is widely grown as an ornamental plant in temperate regions worldwide. In contrast, the subsp. *japonica* is a small shrub that gives an impression of weakness. It is very rare, scattered in deep mountains at elevations of 1000–2000 m, and restricted to specific locations, such as ridges, rocks, and forest margins, making it challenging to cultivate at low elevations in Japan [21,22].

According to our field surveys, the subsp. *japonica* populations in China are predominantly distributed in nature reserves or scenic areas and are less affected by human activities. Its seeds are spread mainly by gravity and water flow over short distances and lack an effective long-distance dispersal mechanism [36]. During the last glacial cycle, they were predicted to have persisted at stable latitudinal distributions by migrating to lower altitudes [23]. A study in Switzerland that examined 26 mountains found that the alpine flora is expanding its range to higher elevations [37]. The fragmentation of the suitable habitat of the subsp. *japonica* may be related to its migration to high-altitude mountains due to climate warming since the Last Glacial Maximum, as well as short-distance dispersal mechanisms.

Habitat fragmentation often leads to a reduction in population size, accompanied by decreased genetic diversity, genetic drift, and inbreeding depression [38]. This can compromise the adaptive capacity of populations, making them highly susceptible to climate change [38,39]. Previous studies have confirmed that populations of the subsp. *japonica* in Japan and Anhui Province, China, generally exhibit low genetic diversity [40,41]. Therefore, habitat fragmentation in the context of climate change makes it more challenging for the subsp. *Japonica* to survival and increases the risk of endangerment.

### 3.3. Distinct Responses of Potentially Suitable Habitats to Future Climate Change in Two Subspecies

**Figure 6:**
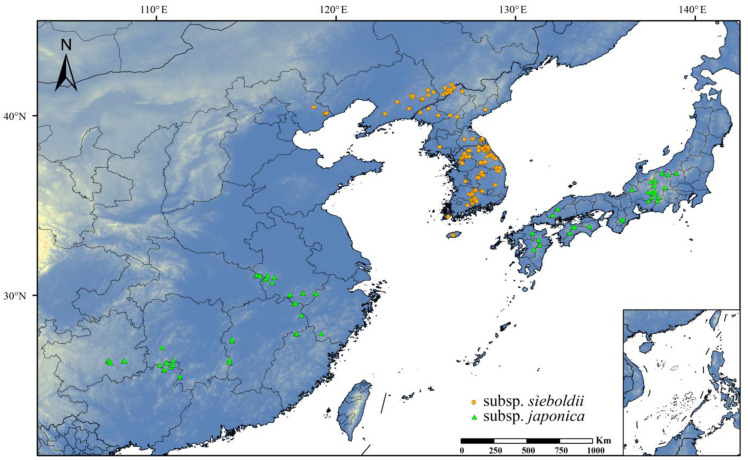
Spatial distribution of occurrence records of subsp. *sieboldii* and subsp. *japonica*.

Based on the changes in centroid analyses, we found that the centroids of highly suitable habitats for the two subspecies tended to move toward higher latitudes in response to climate warming, which is consistent with a previous study predicting that Northern Hemisphere species would shift to higher latitudes with global warming [16]. However, the extent and area of suitable habitats for these two subspecies react differently to future climate change. With the progression of climatic warming, moderately and highly suitable habitats for the subsp. *japonica* are projected to decrease notably, whereas those for the subsp. *sieboldii* are expected to expand northward, leading to a marked increase in their distribution area. This is consistent with predictions for *Platycladus orientalis* across five Chinese seed zones under future climate scenarios [16]. These predictions indicated a general decline in suitable habitats for *P. orientalis* within the subtropical seed zone, in contrast to an expansion in the northern seed zone, where a more pronounced shift toward higher latitudes was observed. Similar results were also found in the prediction of potential habitats for three subspecies of *Cervus nippon* in China [24]. They projected that highly suitable habitats for the subsp*. hortulorum* would progressively expand under climate change, whereas those for the subsp*. kopschi* would steadily decline and potentially vanish by the 2050s under the SSP585 scenario. In species with a widespread distribution, various populations typically encounter unique environmental conditions that foster local adaptations and diverse reactions to climate change [16]. The geographical distribution of *M. sieboldii* spans a large range (107.3–138.9°E, 36.8–40.5°N) (Figure 6) and the distinct responses of its subspecies to climate change may be attributed to the long-term occupancy of specific ecological niches, leading to unique local adaptations.

It is worth noting that our simulations indicated that the current highly suitable habitat for the subsp. *japonica* in China, which is closely aligned with its actual distribution, is expected to gradually decrease and ultimately vanish with climatic warming. This suggests that existing populations may face extinction unless they adapt to the changing climate through natural selection or migrate to habitats suitable for survival after climate warming. However, this assumption is unrealistic, because natural selection relies on random mutations. For many species, the rate of natural selection is slower than that of global warming, which makes it insufficient for rapid adaptation [2]. Migration involves the long-distance dispersal of seeds or other reproductive materials by plants and can lead to competition with local species in new environments, often resulting in the suppression or even extinction of one of the competing species [2]. Furthermore, the fragmentation of natural landscapes and altitude hinders species migration [42]. Given that the subsp. *japonica* is not a dominant species within its community, it faces competitive disadvantages [12], and its seeds lack effective long-distance diffusion mechanisms. Therefore, achieving population sustainability through natural migration remains impractical, even if the suitable habitat (which is mainly poorly suitable) for the subsp. *japonica* continues to increase with the intensification of climate warming, as predicted.

### 3.4. Protection Advice for Two Subspecies

Based on the aforementioned analysis, the subsp. *japonica* should be given priority in conservation efforts over the subsp. *sieboldii*, particularly in the populations present in China. First, monitoring and in situ conservation measures should be implemented for existing wild populations, particularly those in Southern China, which are projected to disappear in the future, in order to maintain the integrity of their ecosystems and habitats. Second, ex situ conservation should complement these efforts by acting as an additional gene bank for wild populations. The predictions suggest that the area at the junction of the Chongqing, Shaanxi, and Hubei Provinces in China will maintain moderate suitability, despite climate change, making it an ideal location for ex situ conservation efforts. Third, initiating the introduction and cultivation efforts for the subsp. *japonica* is crucial because of its high ornamental value. It can be introduced into areas with similar native habitats for ornamental purposes, as it relies on high altitudes, and mountainous scenic areas may be a good choice. These predictions suggest that the provinces of Zhejiang, Jiangxi, and Hunan in China will retain large areas of poorly suitable habitats for the subsp. *japonica* under warming climatic conditions, making them potential regions for introduction. Furthermore, breeding studies aimed at drought resistance, tolerance to high summer temperatures, and adaptation to low-altitude growth should be conducted. In addition, to ensure population stability, actions are required to increase genetic diversity and prevent genetic drift and inbreeding depression [39]. It is advisable to develop a population recovery plan that includes the release of new individuals into existing populations in order to expand their size and gene pools. Our field survey revealed that seedlings from existing populations within the Qingliangfeng National Nature Reserve were successfully cultivated and reintroduced. Hybridization between different regional populations can increase gene flow and boost genetic diversity; however, precautions must be taken to avoid outbreeding depression [39]. Finally, according to our predictions, some highly suitable habitats such as those in central and northeastern Honshu Island, Japan, are expected to remain relatively stable, despite global warming. Therefore, these areas present an opportunity for long-term in situ conservation plans, which can be further supported through extensive international and regional cooperation.

For the subsp. *sieboldii*, although its projected potentially suitable habitats will expand northward with climatic warming, similar to those of subsp. *japonica*, its weak migration ability may limit its natural expansion. Therefore, we should pay attention to the changes in wild populations, conduct resource surveys in potential habitats in the north, and intervene when necessary to ensure migration. The subsp. *sieboldii* is widely grown as an ornamental plant in temperate regions worldwide. Enhancing flower color diversity, improving ornamental value, and utilizing its strong cold tolerance for hybridization with other Magnoliaceae species to develop cold-resistant varieties are promising breeding strategies. Moreover, conservation recommendations for the subsp. *japonica* also apply to the subsp. *sieboldii*, such as in situ and ex situ protection, introduction and cultivation, hybridization among populations in different regions to increase genetic diversity, and international cooperation on conservation.

### 3.5. SDMs in Subspecies Level May Provide More Information 

SDMs can be used to predict habitat suitability under different climate change scenarios based on occurrence at the species level. These results could contribute to the assessment of potential risks and development of conservation strategies for climate change. However, previous studies have suggested that elaborate and independent predictions of habitat suitability should consider different taxa below the species level, such as subspecies, that are distributed across different ecological regions [24]. In our results, the two subspecies (subsp. *japonica* and subsp. *sieboldii*) showed different responses to climate change, and independent consideration of different subspecies will provide more useful information and facilitate targeted recommendations for the conservation and sustainable use of forest genetic resources. Additionally, the results of SDMs may depend on the distinct distribution patterns of two subspecies; the subsp. *sieboldii* exhibited a clearer continuous distribution in Northeast China and the Korean peninsula, while the subsp. *japonica* exhibited a discontinuous distribution between China and Japan, which may lead to discontinuous ecological factors in SDM simulations. However, we collected as many occurrences of the subsp. *japonica* as possible, and the environmental response curves were still not smooth enough (Supplementary Figure S2), which might have resulted in anomalies in the SDM simulations. Therefore, when using SDMs to predict species distributions, it is essential to consider species distribution characteristics, increase the number of occurrence records, or further optimize SDMs to minimize fitting errors [43,44].

## 4. Materials and Methods

### 4.1. Acquisition and Preprocessing of Occurrence and Map Data

The occurrence records of the two subspecies were obtained through field surveys, databases of the Chinese Virtual Herbarium (CVH, http://www.cvh.ac.cn/ (accessed on 1 January 2024)), Global Biodiversity Information Facility (GBIF, https://www.gbif.org (accessed on 1 January 2024)), Chinese Plant Photo Bank (PPBC, https://ppbc.iplant.cn/ (accessed on 1 January 2024)), and relevant literature records. To ensure the accuracy of the data, a field survey of the distribution of *M. sieboldii* in China was conducted. For the subsp. *japonica*, which has few occurrence records, we conducted field surveys at all distribution points we found in China. For the subsp. *sieboldii*, which had more occurrence records, we randomly selected distribution sites to conduct field surveys.

The accuracy and reliability of the occurrence data are directly related to the credibility of the prediction results, and an overly dense distribution of occurrences can lead to the overfitting of the MaxEnt model [32,33,34]. Therefore, the occurrence records were calibrated and verified by removing erroneous, duplicate, and incomplete data. Then, the software ENMTools v.1.1.2 [45] was employed to filter the occurrence records using the spatial resolution (2.5′ × 2.5′) of the climate-data layers (Section 4.2) used in this study. Only records closest to the center of each grid were retained [46]. A total of 185 occurrence records were obtained (Figure 6), including 115 for the subsp. *sieboldii* and 70 for the subsp. *japonica*.

A vector map of the world was downloaded from the official Diva-GIS website (https://diva-gis.org/data.html (accessed on 1 January 2024)).

### 4.2. Acquisition and Preprocessing of Environmental Data

Climatic conditions significantly affect the geographical distribution of plants. Altitude, which is closely associated with temperature, oxygen content, and atmospheric pressure, is equally important for plant growth, development, and distribution [47]. Therefore, we selected 19 climatic variables and altitudes to construct the MaxEnt model. All data were downloaded from the World Climate Database (http://www.worldclim.org/ (accessed on 1 January 2024)), with a spatial resolution of 2.5′ × 2.5′. Baseline climate data (average for the years 1970–2000) were used as current climate data. The BCC-CSM2-MR climate system model data released by CMIP6 were used as the future climate data. We selected data for two periods (2041–2060 and 2081–2100) under two climate scenarios: SSP126 and SSP585. SSP126 represents a scenario with low greenhouse gas concentrations, in which the radiative forcing decreases to 2.6 W/m^2^ by 2100. In contrast, SSP585 represents a composite scenario of an energy-intensive socio-economic development path with strong radiative forcing, in which radiative forcing rises to 8.5 W/m^2^ by 2100 [48].

Judicious selection of environmental variables can prevent overfitting of the training data and enhance the accuracy of the model in predicting species distributions [49,50]. First, 20 environmental variables and occurrence data for the two subspecies were input into the MaxEnt model and run separately. The model parameters were set as follows: output format and output file type were set to “logistic” and “. asc”, respectively; 25% of the occurrence data were set as test data; the jackknife test was used to obtain the percentage contribution and permutation importance of each environmental variable; the model was run 10 times to take the average; and the remaining parameters were set to default. The values of the environmental variables for the 115 and 70 occurrence points were then extracted using ArcGIS v.10.4.1 and imported into SPSS v.20 to perform a pairwise Pearson correlation analysis. Next, variables with a contribution rate of zero were removed according to the jackknife test’s result, and when there was a high correlation between two variables (|r| ≥ 0.8) (Figure S3), only the variable with the highest contribution to the jackknife test was retained [51,52]. Finally, five variables for the subsp. *sieboldii* and seven variables for the subsp. *japonica* were selected for model construction (Table 3).

### 4.3. Model Optimization, Construction, and Evaluation

To improve the prediction performance of the model, the Kuenm package [53] in R v.4.2.0 was used to optimize two parameters of MaxEnt, the RM and FC, following the approach described by Yan et al. [54]. Optimal FC and RM values were then used to predict suitable habitats for the two subspecies under current and future climatic scenarios. The preprocessed environmental variables and occurrence data were input into the MaxEnt model, with the optimal combination of RM and FC set as the parameters. Response curves of the environmental variables were created to illustrate the relationship between species distribution probability and environmental variables. A ROC curve was constructed, and the AUC was used to assess the model accuracy. When the AUC exceeded 0.9, the prediction performance was considered excellent [55]. The other settings were similar to those described in Section 4.2.

### 4.4. Classification of Suitable Habitat

The prediction results generated by the MaxEnt model were transformed into raster data using the conversion tool of ArcGIS v.10.4.1. Then, the grades of suitable habitats were reclassified using the manual classification method as follows: unsuitable habitats (*p* ≤ 0.2), poorly suitable habitats (0.2 < *p* ≤ 0.4), moderately suitable habitats (0.4 < *p* ≤ 0.6), and highly suitable habitats (*p* > 0.6) [55].

### 4.5. Changes in Centroids of Highly Suitable Habitat

Highly suitable habitats were closely aligned with the actual distribution of the species, making the analysis of their trends more significant. To understand the trends in the variation in highly suitable habitats for the two subspecies from the present to the future under different climate scenarios, the migration of their centroids was analyzed. The simulation results generated by the MaxEnt model under different climate scenarios for different periods were imported into ArcGIS v.10.4.1 and converted into binary data. Then, the command “centroid changes” of the SDMtoolbox [56] was employed to determine the position and migration direction of the centroid of the highly suitable habitats and to calculate the migration distance [57].

## 5. Conclusions

In this study, potentially suitable habitats for two subspecies of *M. sieboldii* in the present and future were simulated using the MaxEnt model. Precipitation in the warmest quarter played a crucial role in shaping the potential habitats of both subspecies; however, they exhibited different sensitivities to temperature-related variables and altitude. The subsp. *sieboldii* was more sensitive to temperature seasonality and annual mean temperature, whereas the subsp. *japonica* was more sensitive to altitude, the mean temperature of the driest quarter, and isothermality. In the current climate, the subsp. *sieboldii* is predicted to have larger, more contiguous suitable habitats across northeastern China (Liaoning and Jilin), the Korean Peninsula, and Japan, whereas the subsp. *japonica* occupies smaller, more fragmented habitats scattered across central and western Japan and the southern Chinese mountains. These two subspecies exhibited distinct responses to future climate change. Each grade of potentially suitable habitat for the subsp. *sieboldii* is projected to expand extensively northward over time, with SSP585 promoting a more significant habitat expansion than SSP126. In contrast, moderately and highly suitable habitats for the subsp. *japonica* are projected to undergo significant contractions in Southern China, except for poorly suitable habitats. The centroids of the potentially highly suitable habitats for both subspecies are predicted to shift to higher latitudes. Therefore, we suggest that the subsp. *japonica* should be prioritized in conservation efforts over the subsp. *sieboldii*. Strategies such as in situ and ex situ protection, introduction and cultivation, hybridization among populations in different regions, and international cooperation in conservation are proposed. In conclusion, our study has important implications for the development of precise and tailored conservation strategies for the two subspecies of *M. sieboldii* to cope with the adverse effects of climate change.

## Figures and Tables

**Table 1:** Percent contribution and permutation importance of the environmental variables.

Variable	Percent Contribution (%)	Permutation Importance (%)	Percent Contribution (%)	Permutation Importance (%)
	subsp. *sieboldii*		subsp. *japonica*	
Bio4	49	15.9	-	-
Bio18	47.8	13.2	61.8	90.4
Bio1	1.9	68.3	-	-
Alt	0.6	1.5	11.5	4.9
Bio15	0.6	1.1	-	-
Bio3	-	-	11.1	1.9
Bio9	-	-	7.3	1.4
Bio14	-	-	6.5	0.1
Bio4	-	-	1.7	1.3
Bio2	-	-	0	0

**Table 2:** Optimum values and suitable ranges of the dominant environmental variables.

Variable	Suitable Range	Optimum Value	Suitable Range	Optimum Value
	subsp. *sieboldii*		subsp. *japonica*	
Bio18 (mm)	473.2–855.5	748.2	≥529.5	836.8
Bio4	850.6–1351.8	1186.6	-	-
Bio1 (°C)	4.4–18.2	11.2	-	-
Alt (m)	-	-	709.0–1776.3	1310.7
Bio9 (°C)	-	-	−5.3–6.0	4.7
Bio3	-	-	23.2–29.8	27.3

**Table 3:** Details of the 20 environmental variables and the selected ones for model construction.

Variable	Description	Unit	Subsp. *sieboldii*	Subsp. *japonica*
Bio1	Annual mean temperature	°C	✔	
Bio2	Mean diurnal range	°C		✔
Bio3	Isothermality (Bio2/Bio7) (×100)	/		✔
Bio4	Temperature seasonality (standard deviation×100)	/	✔	✔
Bio5	Max temperature of the warmest month	°C		
Bio6	Min temperature of the coldest month	°C		
Bio7	Annual temperature range (Bio5–Bio6)	°C		
Bio8	Mean temperature of the wettest quarter	°C		
Bio9	Mean temperature of the driest quarter	°C		✔
Bio10	Mean temperature of the warmest quarter	°C		
Bio11	Mean temperature of the coldest quarter	°C		
Bio12	Annual precipitation	mm		
Bio13	Precipitation of the wettest month	mm		
Bio14	Precipitation of the driest month	mm		✔
Bio15	Precipitation seasonality (coefficient of variation)	/	✔	
Bio16	Precipitation of the wettest quarter	mm		
Bio17	Precipitation of the driest quarter	mm		
Bio18	Precipitation of the warmest quarter	mm	✔	✔
Bio19	Precipitation of the coldest quarter	mm		
Alt	Altitude	m	✔	✔

Variables marked with “✔” were ultimately selected for constructing the MaxEnt model.

## Data Availability

The original contributions presented in the study are included in the article/Supplementary Materials; further inquiries can be directed to the corresponding authors.

## Supplementary Materials

The following supporting information can be downloaded at https://www.mdpi.com/article/10.3390/plants13213097/s1, Figure S1: Response curves of the dominant environmental variables for subsp. *sieboldii*; Figure S2: Response curves of the dominant environmental variables for subsp. *japonica*; Figure S3: Correlation between 20 variables (subsp. *japonica* is above the diagonal, subsp. *sieboldii* is below the diagonal); Table S1: Changes in the area of suitable habitats of the two subspecies in different periods under two climate scenarios.
