# Supplementary figures and images for: Distinct Ecological Habits and Habitat Responses to Future Climate Change in Two Subspecies of Magnolia sieboldii K. Koch, a Tree Endemic to East Asia
Source: Plants (Basel). 2024 Nov 3;13(21):3097. doi: 10.3390/plants13213097 (PMC11548506; doi:10.3390/plants13213097)

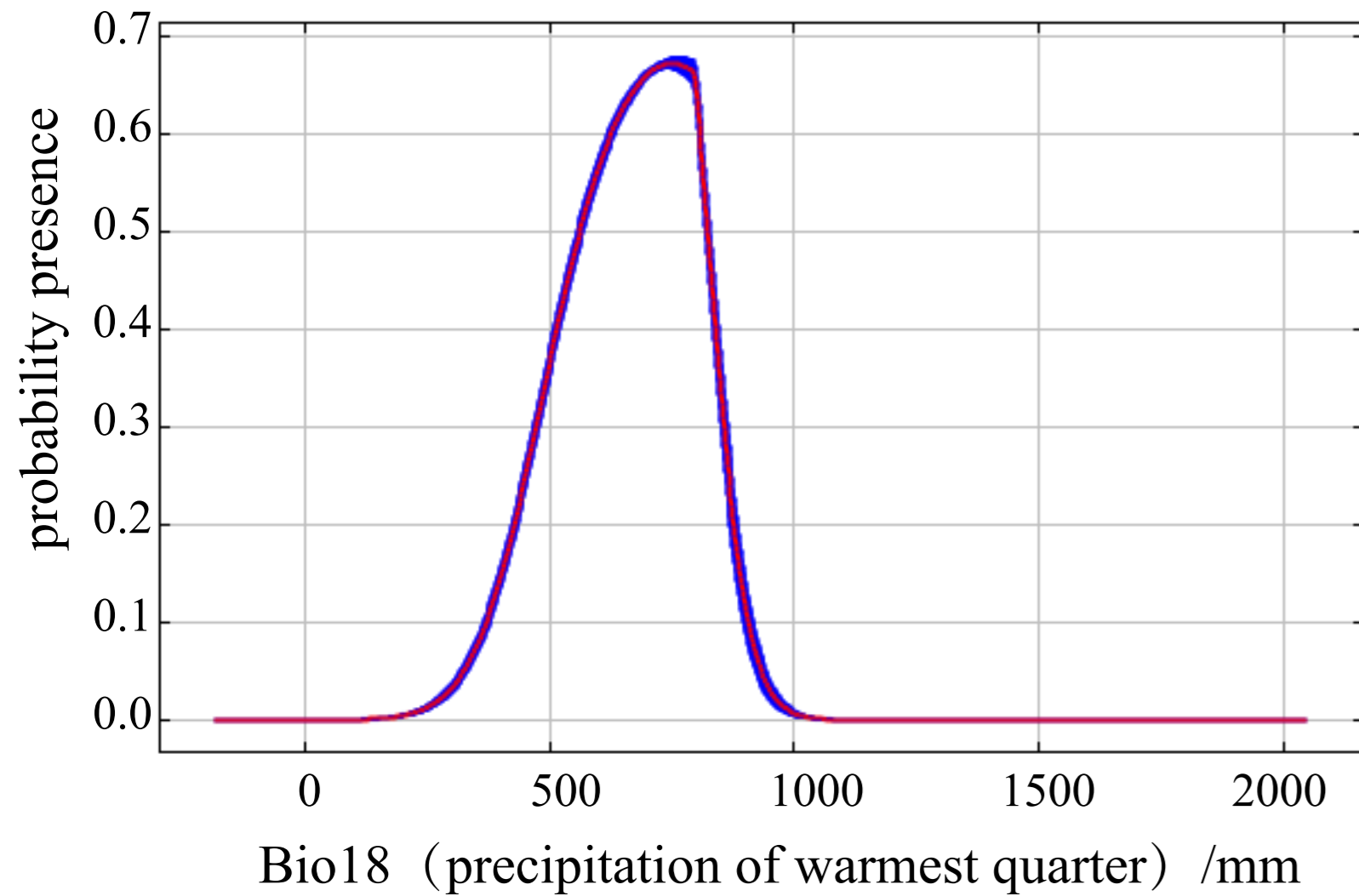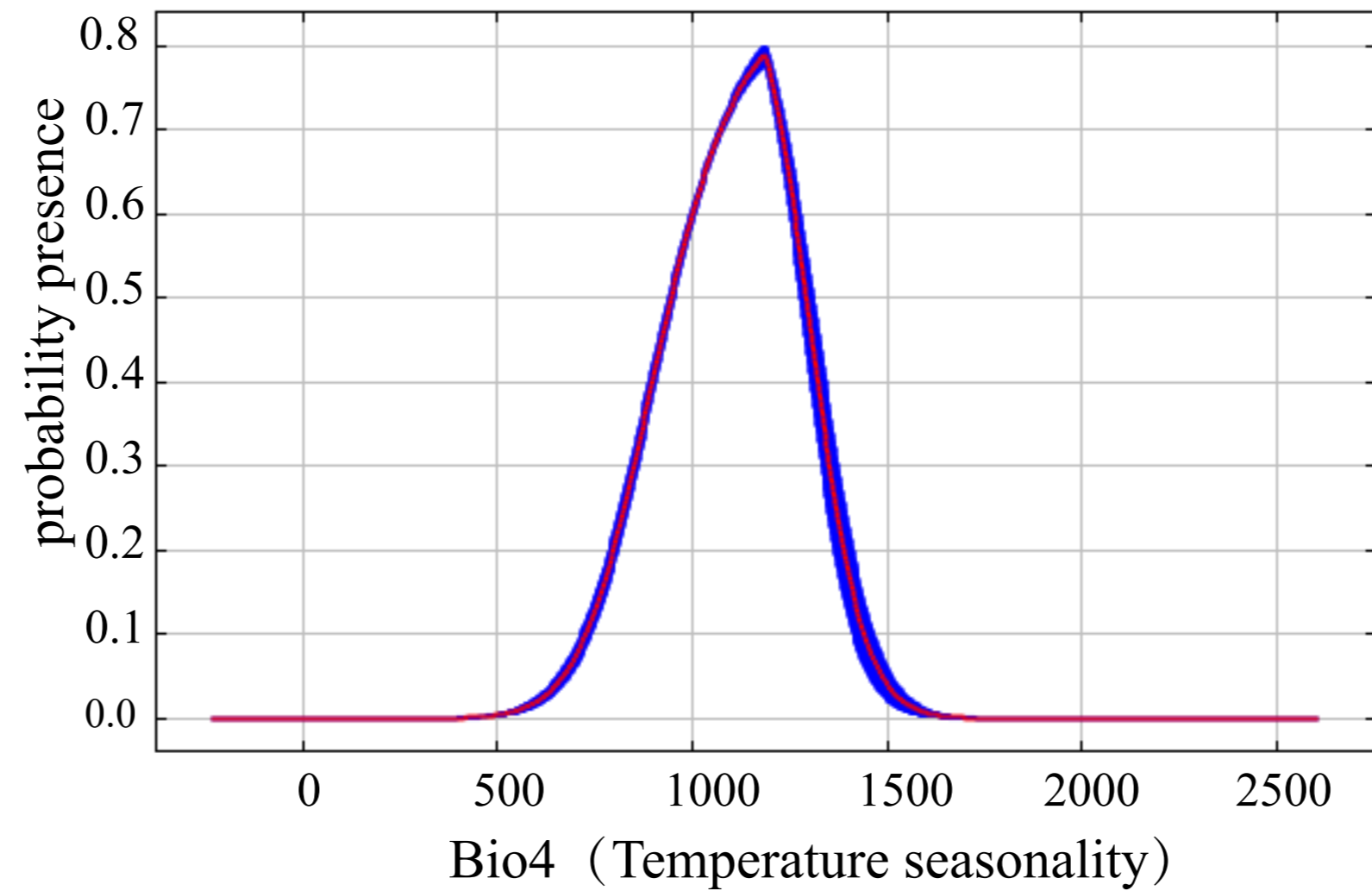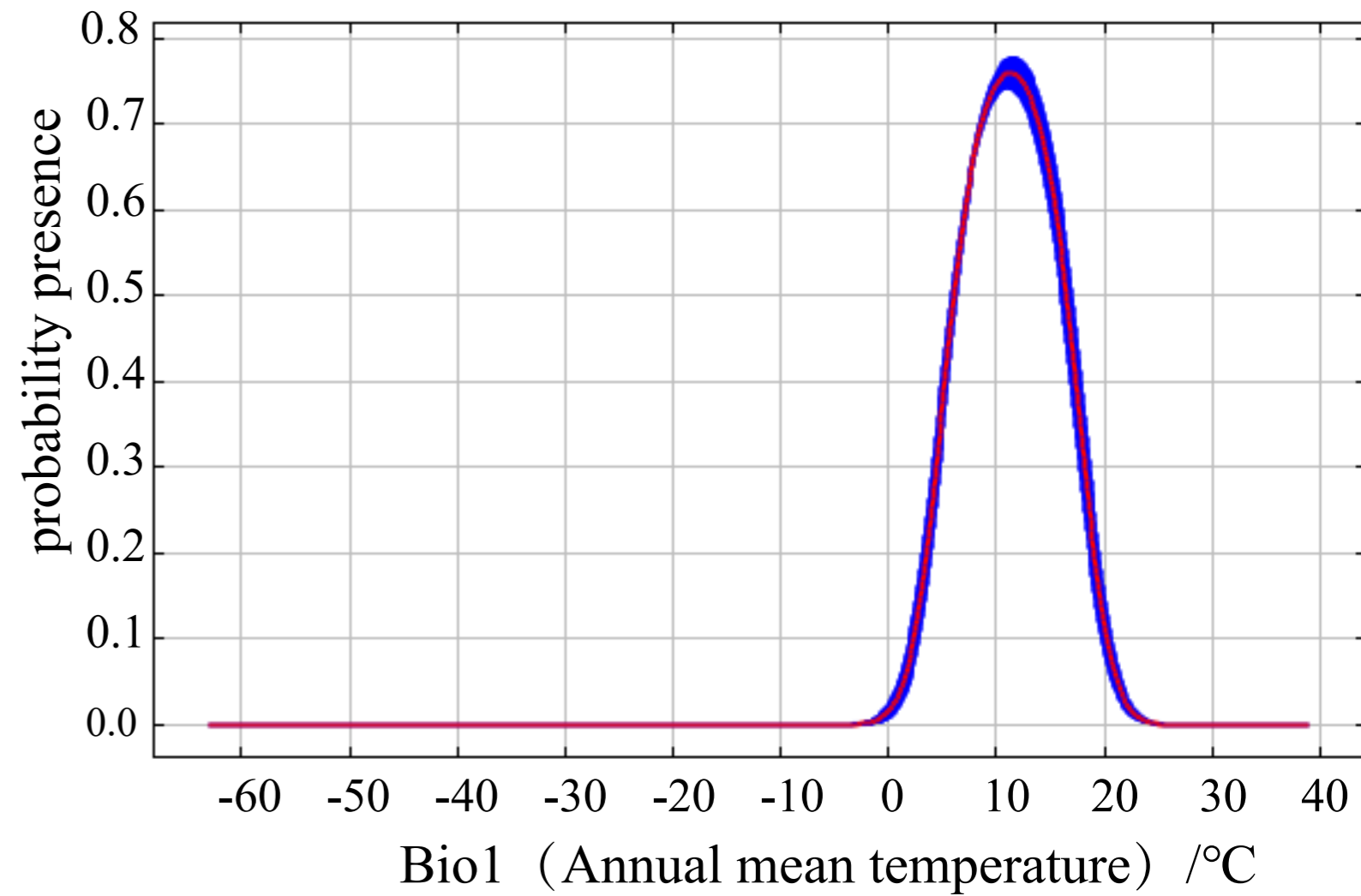

Supplement: Supplementary file 1 [file plants-13-03097-s001.zip › Figure S1 Response curves of the dominant environmental variables for subsp. Sieboldii.pdf]

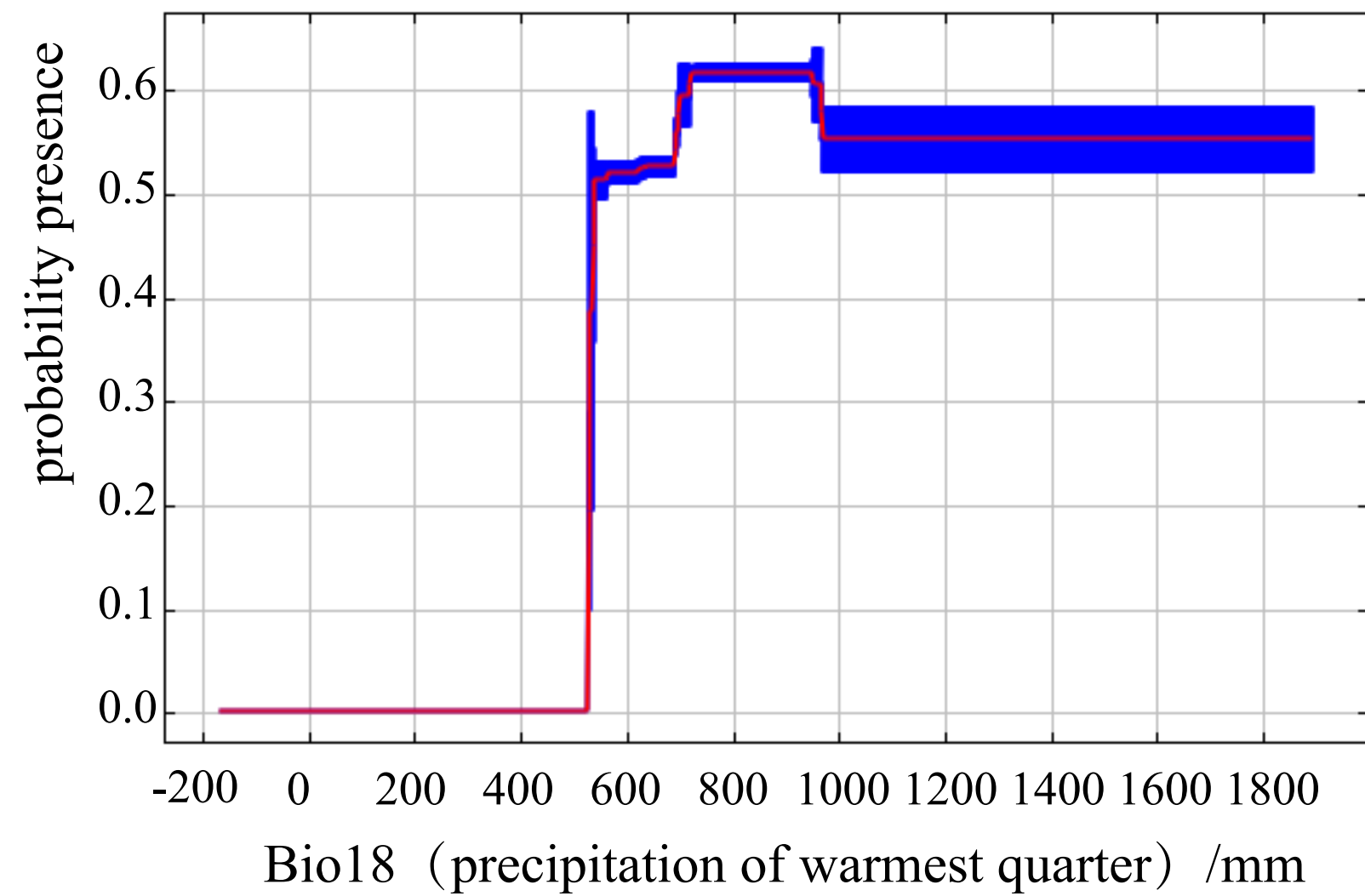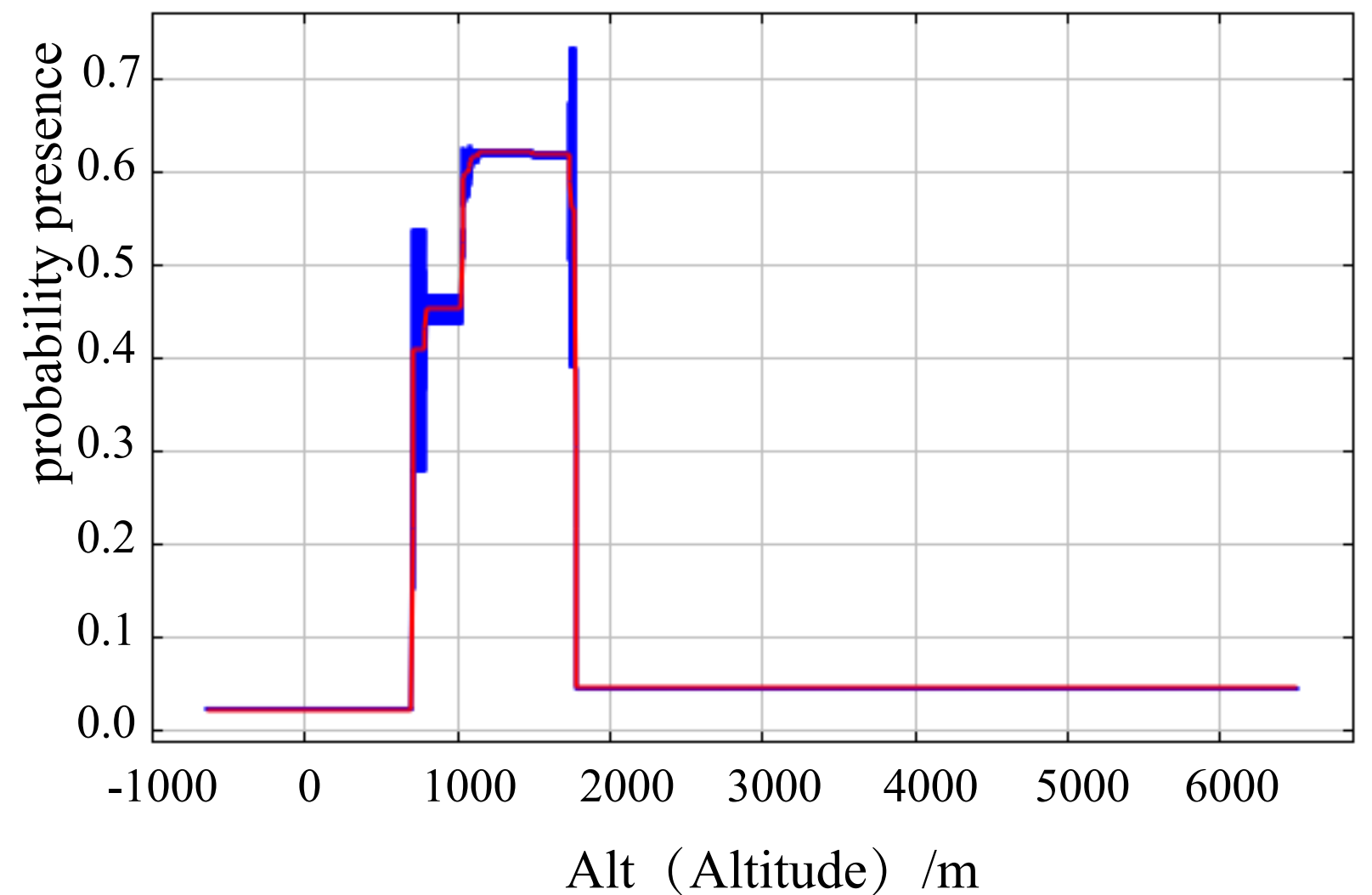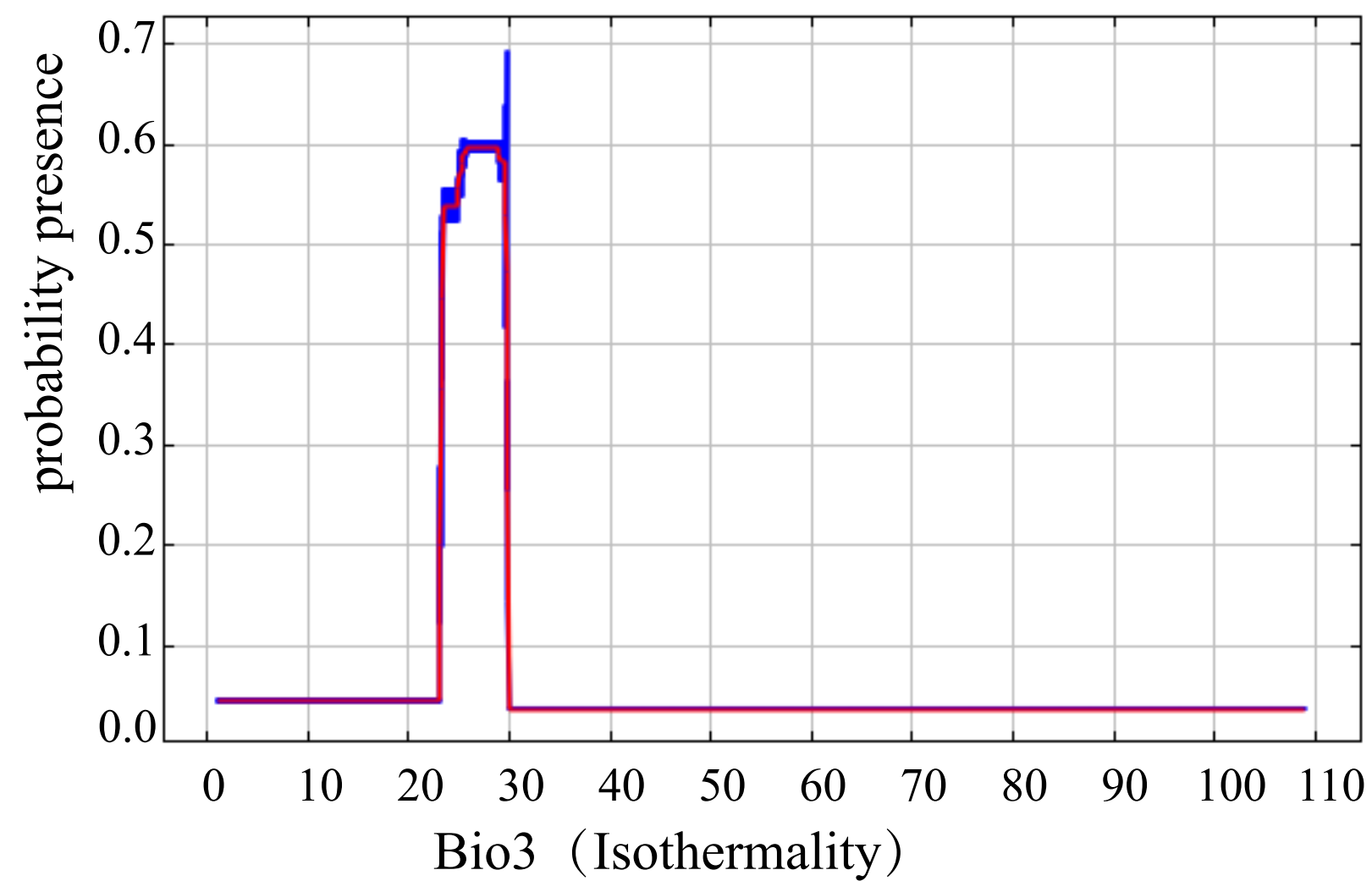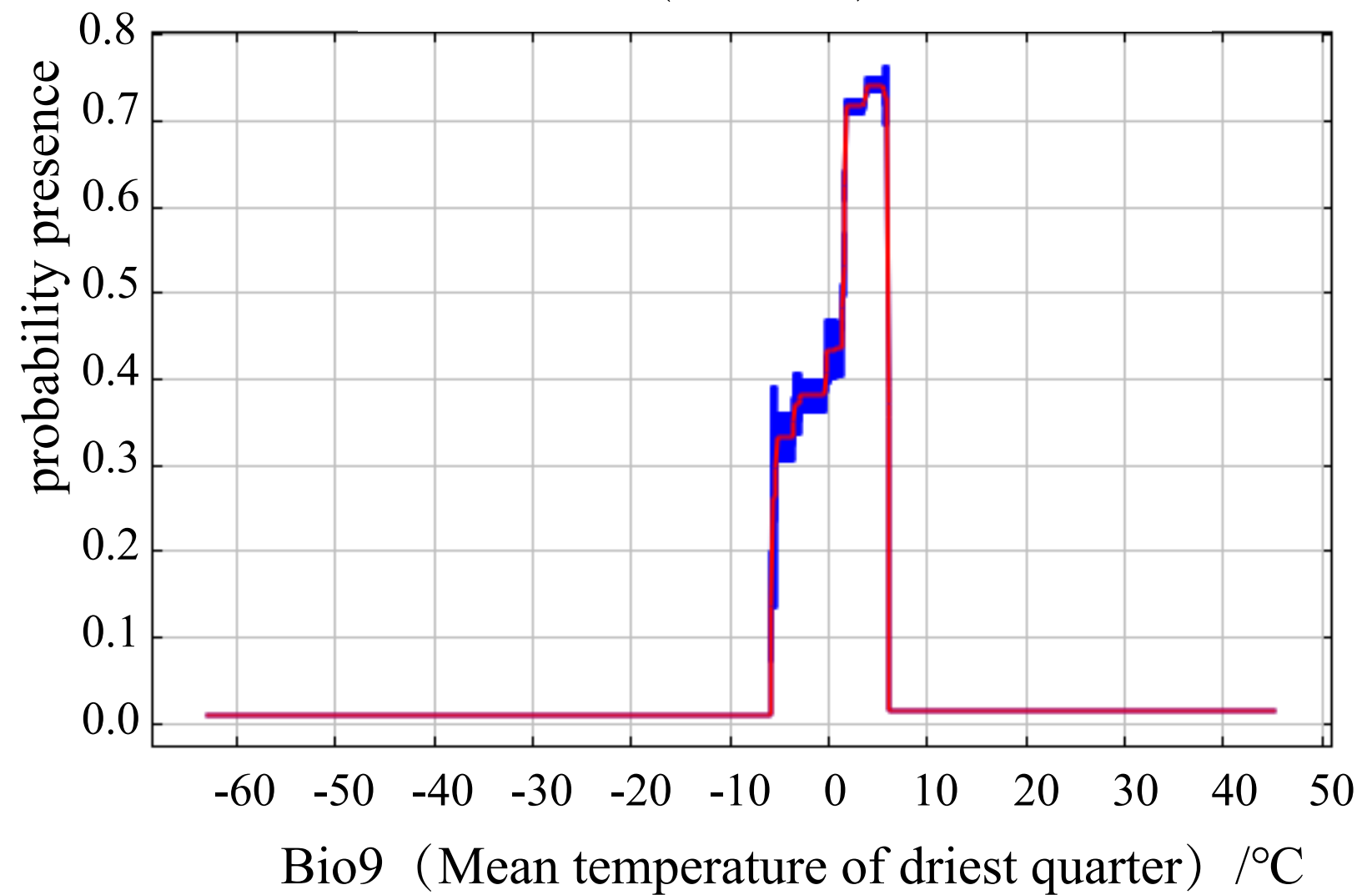

Supplement: Supplementary file 1 [file plants-13-03097-s001.zip › Figure S2 Response curves of the dominant environmental variables for subsp. japonica.pdf]

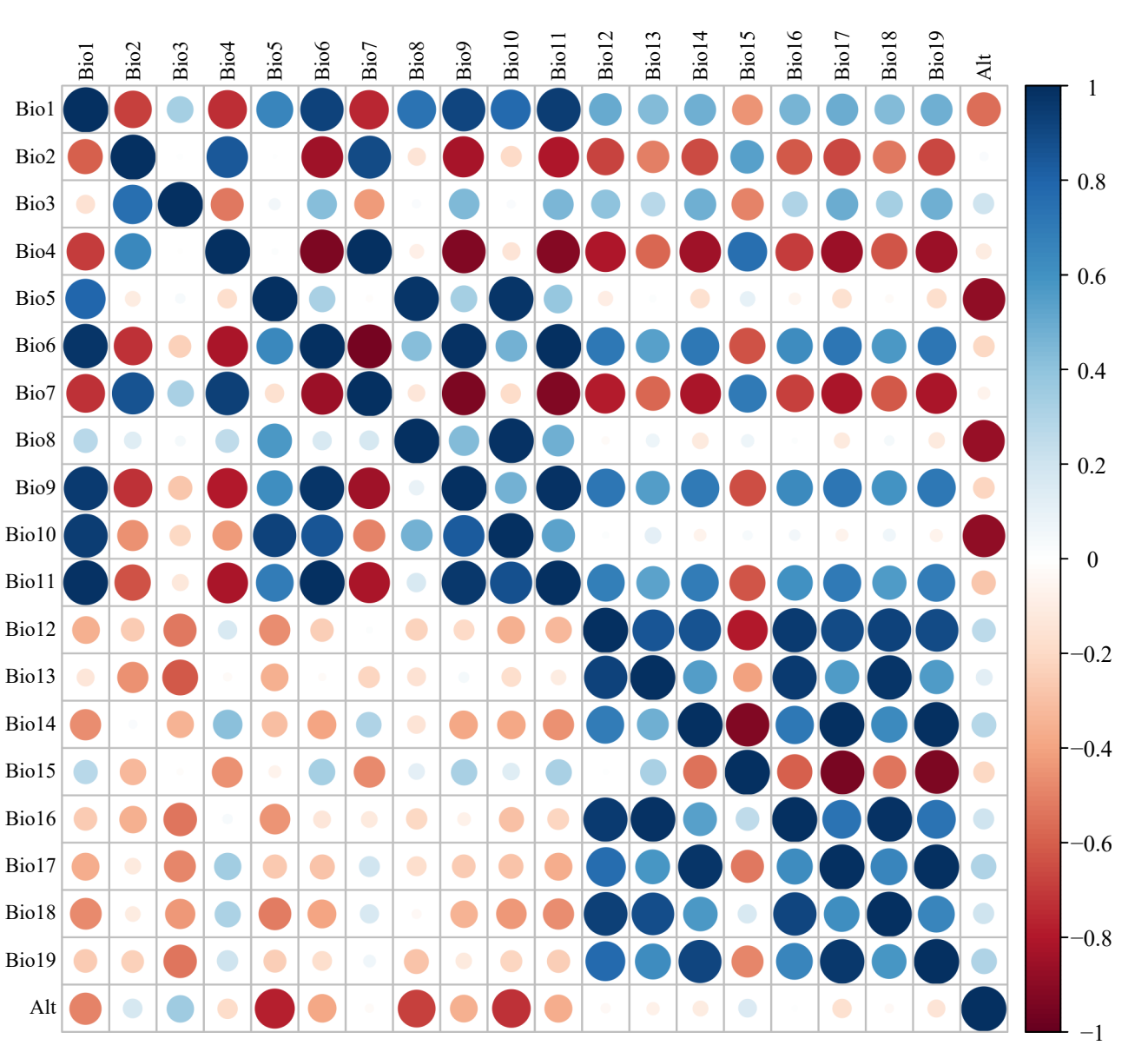

Supplement: Supplementary file 1 [file plants-13-03097-s001.zip › Figure S3 Correlation between 20 variables (subsp. japonica is above the diagonal, subsp. sieboldii is below the diagonal).pdf]
